# Hop Phytochemicals and Their Potential Role in Metabolic Syndrome Prevention and Therapy

**DOI:** 10.3390/molecules22101761

**Published:** 2017-10-19

**Authors:** Pavel Dostálek, Marcel Karabín, Lukáš Jelínek

**Affiliations:** Department of Biotechnology, University of Chemistry and Technology, Prague, Technická 5, 166 28 Prague 6, Czech Republic; Marcel.Karabin@vscht.cz (M.K.); Lukas.Jelinek@vscht.cz (L.J.)

**Keywords:** hops, xanthohumol, iso-α-acids, matured hop bitter acids, metabolic syndrome, obesity, diabetes, cholesterol

## Abstract

Historically, hop cones (*Humulus lupulus*) have been used since ancient times as a remedy for many ailments and, as a source of polyphenols and bitter acids, is very effective in the treatment of metabolic syndrome (MS). Hop flavonoids, particularly xanthohumol (XN), are substances with hypoglycemic, antihyperlipidemic, and antiobesity activities. Iso-α-acids (IAA) and matured hop bitter acids (MHBA) improve health by influencing lipid metabolism, glucose tolerance, and body weight. The modulatory effect of IAA and MHBA on lipid metabolism may also be responsible for a loss in body weight. These results suggest promising applications for IAA, MHBA, and XN in humans, particularly in the prevention of diet-induced obesity and diabetes.

## 1. Introduction

Hop is a perennial and dioecious climbing plant and only female infertile plants are cultivated to produce hop cones. Hop cones consist of a strig and bracts with glands that produce yellow lupulin. Hop cones are nowadays processed for pressed hops, hop pellets, and hop extract (extraction with ethanol or supercritical CO_2_) and other isomerized or fractionated products. Most hops are used today in beer production. From the point of view of content, hop resins, hop polyphenols, and hop essential oils are important for brewers. Isomerised products of hop resins give beer bitterness, hop polyphenols promote the precipitation of proteins, and hop essential oils give the beer a distinctive hops flavour. Hop (*Humulus lupulus* L.) belongs to the same family as hemp (*Cannabaceae* family) and, as expected, contains a wide range of secondary metabolites [1,2]. In addition to the variety, they are dependent on the growing habitat and its climatic conditions [3].

In ancient times, hops were used as a remedy against many ailments [4,5]. This function of hop, particularly its anti-inflammatory effect, was mentioned in a book from the 11th century attributed to the Arabic doctor Mesue, and Hildegard von Bingen, the German botanist (1158), reported that hop suppresses putrefaction in beverages [6]. During the Renaissance period, treatment with hops became more widespread. Paracelsus used hops against indigestion and Matthiolus, the doctor and botanist who lived in Prague, proposed to use hops due to their diuretic activity and ability to increase the secretion of bile [6]. During 19th century hop drops were used for treatment of sleeplessness [6,7]. Frequently, and up to the present time, a mixture of hop extract and an extract from *Valeriana officinalis* was used for its sleep improving effects [8]. However, within 50 years, another effect of hop—the estrogenic effect, was discovered [4], although it took longer before the active substance, 8-prenylnaringenin (8-PN), was identified the strongest phytoestrogen in the plant kingdom [9]. Based on this knowledge, there are currently a number of dietary supplements on the market containing standardized 8-PN content for suppression of menopausal symptoms [5]. One substance with a very wide spectrum of activities was rediscovered in beer in 2002, during screening of substances with anticancer potential [10,11,12], although the structure of this substance, the prenylated chalcone, xanthohumol (XN), was resolved by Verzele et al., in 1957, 60 years ago [13]. There are now many papers describing XN and its biological activities [4,5,11,12,14,15,16]. Technologies for the isolation of XN and enrichment of XN in beer have also been developed [17,18,19]. Mainly due to this substance, hop is considered as one of the most promising natural sources of biologically active substances [11,12].

**Figure 1:**
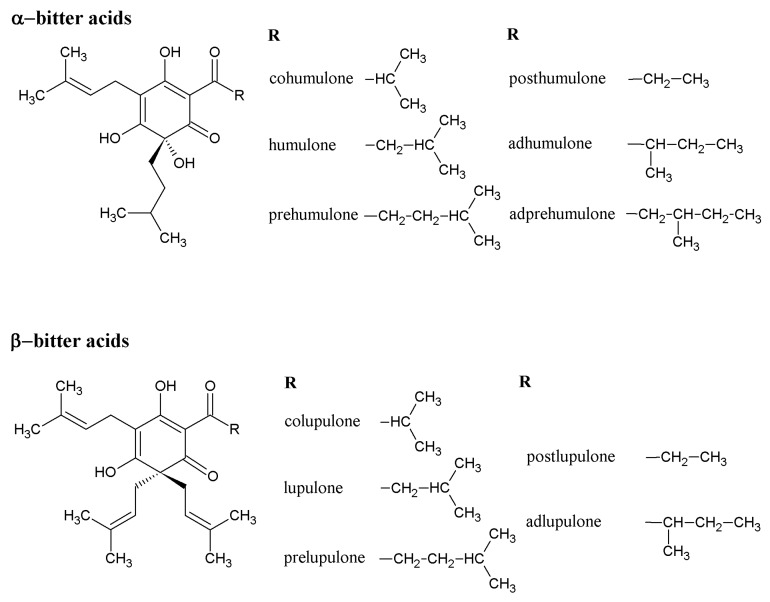
Chemical structures of main hop bitter acid analogues: α-bitter acids and β-bitter acids.

**Figure 2:**
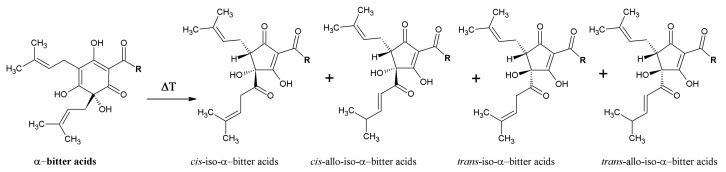
Isomerization of hop α-bitter acids.

**Figure 3:**
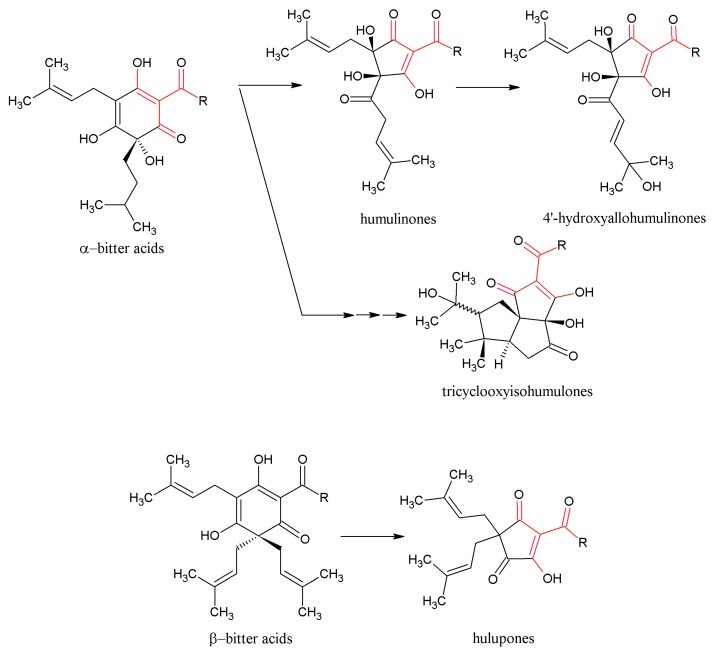
Schema oxidation of α-bitter acids (AA) and β-bitter acids (BA) to matured hop bitter acids (MHBA)—containing β-tricarbonyl moieties (pointed by red colour).

The α-bitter acids occur in soft resins and represent a key component of hop resins. α-Acids (AA) are present in lupulin particles inside the hop cones [3]. The α-bitter acids consist of three major analogues (Figure 1) that are present in all hop varieties: adhumulone, cohumulone, and humulone [3,4,20]. There are also three minor analogues of AA, posthumulone, prehumulone, and adprehumulone (Figure 1) [20]. Isomerization of AA takes place in the brewhouse during hop boiling and results in the formation of their corresponding iso-α-bitter acids (IAA) (Figure 2), which originally occur in *cis* and *trans* forms [20,21]. IAA are very sensitive to oxidation [22]. The exact stereochemistry of *trans* and *cis* IAA substances was determined in 1971 [23], although unfortunately, not correctly. For this reason, all older textbooks contain the wrong structure of *trans* and *cis* IAA [3,20], which was corrected by re-evaluation of the stereochemical structure by Urban et al. in 2013 [24]. The β-bitter acids (BA) (Figure 1) which are also components of soft resins, consist of similar analogues as in case of AA: adlupulone, colupulone, lupulone, postlupulone, and prelupulone [3,4,20]. These substances have very limited solubility in water and for this reason, have low importance in the brewing process. On the other hand, their antimicrobial activity is very strong and nowadays they represent an alternative to antibiotics for the eradication of *Helicobacter pylori* [25]. Recent research has focused on elucidation of molecular structures that are created by oxidation of AA and BA [26,27,28]. These oxidized substances are more hydrophilic than AA and BA and their bitterness is substantially lower than IAA. These structures, containing β-carbonyl moieties, are shown in Figure 3. The group of these substances is called [28] matured hop bitter acids (MHBA). These substances were originally found in storage hop, but technology now exists for the preparation of MHBA from hop extract [28].

**Figure 4:**
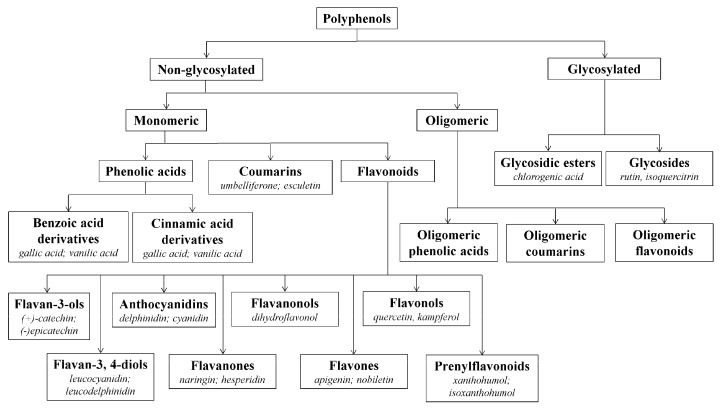
An overview of the hop polyphenols.

**Figure 5:**
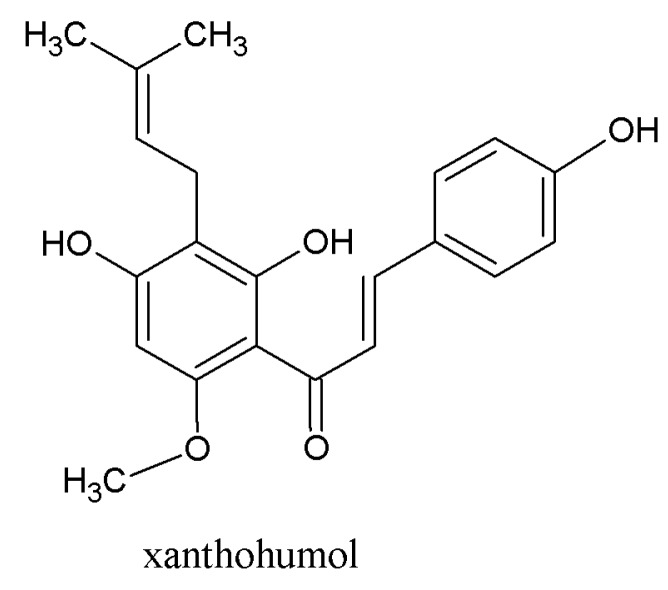
Xanthohumol (XN).

**Figure 6:**
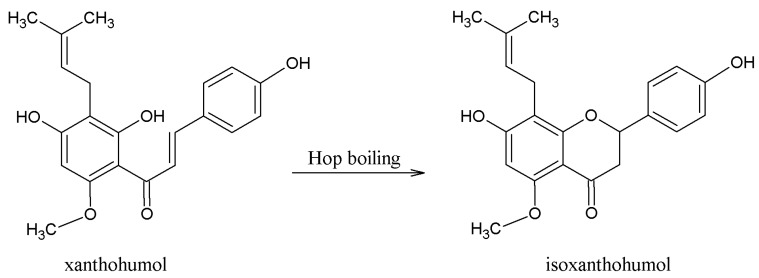
Isomerization of xanthohumol (XN) to isoxanthohumol (IXN) during hop boiling.

Hop polyphenols represent a very broad and heterogeneous group of secondary metabolites with very different chemical structures [3]. About 1000 polyphenolic substances have been found in hop cones. These polyphenols account for about three to eight percent of dry hop cones. Polyphenols are generally found in the green part of the cone. Only prenylflavonoids are present, together with hop resins and essential oils in lupulin granules [29]. We can divide the hop polyphenols into 2 groups (Figure 4): non-glycosylated and glycosylated polyphenols. Non-glycosylated polyphenols consist of monomeric and oligomeric forms. Major oligomeric hop flavonoids are called tannins. Hop monomeric acids consist of phenolic acids (gallic acids, vanillic acids), coumarins (umbeliferone, esculetin), and flavonoids. The flavonoid group consists of flavan-3-ols ((+)-catechin, (−)-epicatechin), anthocyanidins, flavonols (quercetin, kaemferol), flavanonols, and prenylflavonoids (isoxanthohumol, XN). Xanthohumol, more precisely, belongs to the group of prenylated chalcones [29]. Major glycosylated polyphenols are from group of glycosides (rutin, isoquercitrin). Rutin and isoquercitrin are glycosylated quercetins (α-l-rhamnopyranosyl-(1→6)-β-d-glucopyranose and quercetin 3-*O*-β-d-glucopyranoside). Most publications over the past 15 years have concentrated on hop polyphenols and prenylflavonoids. This is mainly because of their health effects [5,10,30]. The most important prenylflavonoids of hop are XN (Figure 5), isoxanthohumol (IXN), and desmethylxantohumol (DMX). Hop can contain about 1% (by weight) of XN [15], but XN is very hydrophobic and its solubility and content in wort, and then beer, is very low. It is interesting that XN is more soluble in dark wort (beer). In addition, xanthohumol becomes isomerized to IXN during hop boiling (Figure 6) [31].

## 2. Principle and History of Metabolic Syndrome

Although the term “metabolic syndrome” is more or less a modern construct, nowadays mainly associated with the development of civilization-related diseases, its history is much longer and more complex. The first references to the common occurrence of elevated blood pressure, hyperglycemia, and hyperuricemia appeared in the 1920s [32]. Later, the issue of fat distribution and its relationship to the predisposition of atherosclerosis and diabetes [33] came to the center of interest in connection with the first evidence that some types of diabetes are not related to insulin secretion but to insulin resistance [34]. The synaptic syndrome (originally syndrome X) [35] for the condition combining the risk of obesity, insulin resistance, and high blood pressure gradually developed at the end of the 20th century [36].

WHO officially defined metabolic syndrome (http://apps.who.int/iris/handle/10665/66040) in 1999. Together with increased knowledge about the importance of individual risk factors, the individual values were modified and their parallel criteria were created by other bodies such as the European Association for the Study of Insulin Resistance (EGIR), the National Cholesterol Education Program (NCEP: ATPIII), the American Association for Clinical Endocrinology (AACE), and the International Diabetes Federation (IDF). Their purpose was to enable the identification of a group of people with an above-average risk of developing type 2 diabetes mellitus and/or cardiovascular disease based on a combination of three of the five risk factors: insulin resistance, high blood glucose, triglycerides/HDL cholesterol concentration, abdominal obesity, high blood pressure. Differences in the levels of the individual parameters led to the unification of methodologies in 2009, when insulin resistance was excluded from prerequisites and the obesity parameters were specified by gender and ethnic origin [37,38].

Based on these definitions, studies defining the prevalence of MS in some countries of the world [37] were developed. It was found that, for example, in the United States, the prevalence of MS in the population exceeds 30% in both men and women and was only slightly lower in Australia. Similar studies have been conducted in European countries, for example, in Denmark and Ireland, where the incidence rates were around 20%. The lower incidence of MS in countries such as China and South Korea is of specific interest because it affects about twice as many women as men. But even in India, the percentage of individuals with MS approaches 20% [37,39].

It has been shown that the occurrence of metabolic syndrome significantly increases the incidence of a wide range of other diseases and thus has mortality and other related socio-economic impacts. At various stages of life, in various orders and with varying degrees of severity, due to a combination of genetic, nutritional, and environmental factors, body function disruption has been shown to be mainly due to changes in glycogen, lipid metabolism, cardiovascular activity, endothelial dysfunction, hormonal changes, increased cell proliferation, and many other factors. Whether the emergence and development of MS is in addition to eating disorders and lack of movement, and whether it is also subject to genetic predisposition is still not completely clear [40], along with relationships between high density lipoprotein content and diabetes.

The incidence of metabolic disease in European countries and the USA has achieved epidemic proportions, with cardiovascular complications and mortality. A metabolic syndrome is associated with five metabolic abnormalities: central obesity, elevated plasma glucose, high concentrations of serum triglycerides, low concentrations of high-density lipoproteins, and elevated blood pressure [41,42]. Therapy is based on the treatment of diabetes and a reduction in the risk of heart disease by decreasing LDL cholesterol and reducing high blood pressure. Of great importance is to reduce weight by appropriate dietary control and exercise [41,43]. Many kinds of diets were tested [44] and it is clear that replacement of refined carbohydrates with proteins, a reduction in saturated fats and an increase in omega-3 oils [44] are very positive. All these requirements are met in a Mediterranean diet that is low in refined carbohydrates and has been shown to reduce HDLs and oxidized low density lipoproteins in women with MS [45]. Positive effects of the Mediterranean diet on MS are based on low levels of refined carbohydrates and saturated fats, a high content of fiber, polyunsaturated oils, and particularly a high content of phytochemicals (such as polyphenols and others) [45,46,47,48,49,50,51,52]. Recently, analysis of data from many studies confirmed that there is a relationship between dietary patterns and MS [53]. The results of another study on the dietary intake of flavan-3-ols proved that this reduced the risk of hypertension in the South Korean adult population [54]. Supplementation of the diet with polyphenols [55], and particularly flavan-3-ols [56], clearly improves MS risk factors.

## 3. Beneficial Effects of Hop Phytochemicals on Metabolic Syndrome

### 3.1. Beneficial Effects of Hop Polyphenols

Hop polyphenols enter the wort during hop boiling and then continue to beer. It is estimated that about 30–40% of polyphenols are from hops. Other polyphenols are derived from malt or unmodified cereals. Another route by which these polyphenols can reach the human population is by the use of hop extracts or hops in the production of food supplements for polyphenols. Most hop polyphenols are similar to polyphenols from other sources, with the exception of XN and other prenylated chalcones that are characteristic of hops [3]. Beneficial effects of polyphenols include hypoglycemic, antihyperlipidemic, and antiobesity activities [4].

The hop flavonoid quercetin was evaluated as an inhibitor of phosphatidylinositol 3-kinase (PI3K). Catechin, epicatechin, quercetin, and rutin were proven to increase insulin secretion in vivo and in vitro by modulation of β-cell proliferation. These polyphenols can act as activators of adenosine 5′-monophosphate activated protein kinase (AMPK) and peroxisome proliferator-activated receptor gamma (PPARγ) [57]. Flavonoids can also act as incretin boosters. Incretins are peptide hormones that control secretion of insulin. Inhibition of α-glucosidase (an enzyme that controls blood glucose levels) by XN was investigated by Liu et al. [58], who demonstrated the positive effect of high levels of polyphenols on the activity of insulin signaling components. Very positive effects of phenolic compounds, particularly flavonoids, for diabetic patients include better control of blood glucose levels and lipid profiles, and a reduction in insulin resistance [59,60,61,62].

Dyslipidemia is defined as a state represented by increased low-density lipoproteins (LDL), very low-density lipoproteins (VLDL), and triglycerides (TG), and decreased levels of high-density lipoproteins (HDL). Oxidation of LDL represents the first stage of atherosclerosis. Under normal conditions, insulin activates the enzyme lipoprotein lipase (LPL), but if insulin is not present, LPL is not activated, resulting in hyperglyceridemia and hypercholesterolemia. This state is very often associated with increased levels of inflammatory mediators such as tumor necrosis factor alpha (TNF-α) or interleukin-6 (IL-6). Hop flavonoids decrease LDL levels and inhibit their oxidation [63]. Antioxidant properties of hop polyphenols are closely linked to their ability to modulate metabolism with a resulting reduction in obesity and weight [59,63]. Catechin, quercetin, and XN can also decrease adipose inflammation in fructose-fed rats and 3T3-L1 adipocytes [64,65].

Xanthohumol has strong anti-obesity activities, which was demonstrated by inhibition of rat liver diacylglycerol acyltransferase and triglyceride transport using HepG2 cells, and secretion of apolipoprotein B [66,67]. Moreover, XN also inhibits cholesteryl ester transfer protein activity, inhibits differentiation of preadipocytes, and induces apoptosis in mature adipocytes [68]. Positive effects of dietary XN on glucose metabolism in male obese rats were detected by Legette et al. (2013). Xanthohumol, in this case, also decreased the risk of hypercholesterolemia and dyslipidemia [42]. Fundamental clinical trials for XN and 8-PN have already been carried out [69,70].

### 3.2. Beneficial Effects of Hop IAA and MHBA

It is interesting that mechanisms of MS affected by hop polyphenols are very similar for IAA [71,72,73,74]. IAA activates peroxisome proliferator activated receptors (PPAR) α and γ in vitro and decreases plasma glucose and lipids in diabetic mice. Expression of PPAR-α was highest in tissues that oxidize fatty acids at a rapid rate. PPAR-γ was mainly present in adipose tissue and was shown to regulate fatty acid storage and glucose metabolism. Diabetic mice in this study had lower plasma glucose and triglyceride levels than the control group [75,76,77,78,79,80]. A study was also carried out with human subjects and, after treatment with 32 mg or 48 mg IAA over a four week period, fasting blood glucose was decreased in comparison with a placebo group [81].

The disadvantage of IAA is its high bitterness. If IAA is required to be added to beverage as a supplement it is better applied as MHBA, which are much less bitter [82,83,84,85]. Application of MHBA was tested on mice models that were fed a high fat diet (HFD). The mechanism of action was also tested. It was discovered that MHBA increased thermogenesis in the brown adipose tissue. Increase heat production is a very elegant mechanism to increase fat burning and could possibly be achieved by diet supplementation (IAA or MHBA). Due to the fact that MHBA is not as bitter and is also not toxic, a double-blind trial was carried out, including a placebo-controlled parallel study with 200 human subjects. A significant reduction in visceral fat area after eight and twelve weeks was recorded. This study confirmed that continual supplementation of MHBA safely reduces body fat, mainly abdominal visceral fat. Results from these studies are very promising and continuous supplementation by IAA or MHBA in humans may help solve obesity [75,76,82,85]. Nevertheless, until now only a limited number of clinical human studies has been done with IAA [74,81] and MHBA [85].

## 4. Conclusions

Hop cones have been used in brewing for many centuries and maybe even more extensively were used in folk medicine. Over this extended period of time, no toxicity was detected. On the other hand, over the last twenty years, hop has been subjected to many studies and several new hop-based activities were discovered. Many papers were published about the relationship between IAA, lipid metabolism, and glucose tolerance, and the IAA effect on body weight reduction and fat reduction was reported. The same effect was also described for MHBA, which are less bitter than IAA. Probably the most interesting substance from hop is XN. This molecule inhibits adipogenesis, increases cell apoptosis, and may have a role in preventing obesity. In our opinion, in the near future, supplementation trials using hop extracts containing both hop polyphenols (preferable XN) and hop IAA or MHBA will be carried out. Fundamental clinical trials for XN and 8-PN have already been carried out. The first real drug based on hop substances should be on the market soon.
